# A Case of Primary Intracranial Sarcoma, DICER1-Mutant, in a Child with a Germline *DICER1* Mutation

**DOI:** 10.3390/brainsci13071040

**Published:** 2023-07-08

**Authors:** Suzanne Elizabeth Kosteniuk, George Michaiel, Christopher Dunham

**Affiliations:** 1 Department of Pathology and Laboratory Medicine, University of Calgary, Calgary, AB T2N 4Z6, Canada; 2 Division of Hematology/Oncology/BMT, Department of Pediatrics, British Columbia Children’s Hospital, Vancouver, BC V6H 3N1, Canada; george.michaiel@cw.bc.ca; 3 Division of Anatomic Pathology, British Columbia Children’s Hospital, Vancouver, BC V6H 3N1, Canada; cdunham@cw.bc.ca; 4 Department of Pathology and Laboratory Medicine, University of British Columbia, Vancouver, BC V6T 1Z7, Canada

**Keywords:** *DICER1*, tumor predisposition syndrome, sarcoma, neuropathology, pediatric neuro-oncology, ICE chemotherapy

## Abstract

*DICER1* syndrome is a tumor predisposition syndrome caused by abnormal micro-RNA processing which leads to a variety of benign and malignant neoplasms in many organ systems, including the central nervous system. This paper reports the case of a primary intracranial sarcoma, *DICER1*-mutant, in a patient with a germline *DICER1* variant thought most likely to be de novo. The patient is a ten-year-old boy who presented acutely with altered level of consciousness, emesis, and left-sided weakness. Imaging revealed a large right frontal hemorrhagic lesion, which was urgently debulked. Histology demonstrated a high-grade sarcomatous lesion. Molecular studies revealed compound heterozygous *DICER1* variants (a frame shift insertion and a missense mutation), and a KRAS missense mutation. The final pathologic diagnosis was rendered to be “primary intracranial sarcoma, *DICER1*-mutant”. Germline genetic testing revealed that the patient possessed a germline *DICER1* variant (parental testing was negative). A dramatic reduction in tumor size was precipitated via chemotherapy (ifosfamide, carboplatin, and etoposide) and radiotherapy (focal proton beam therapy). There was no evidence of residual disease at the primary site at the end of the therapy.

## 1. Introduction

**Figure 1:**
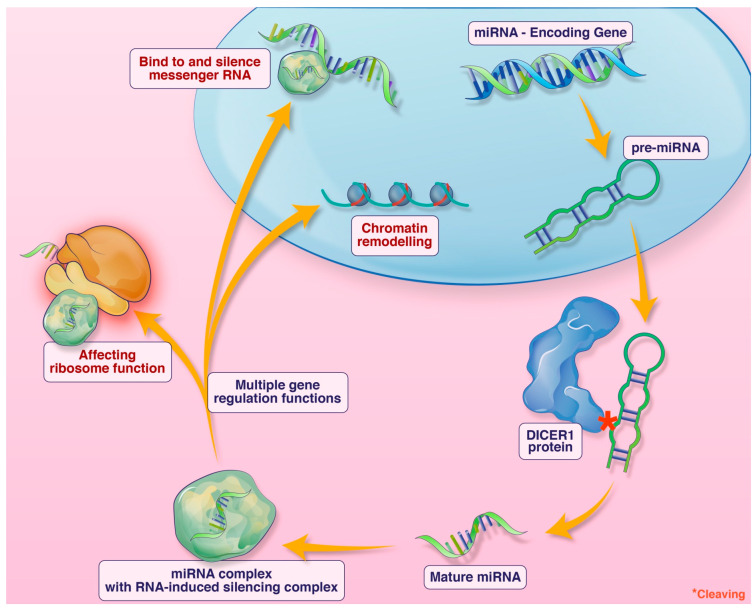
An illustration of the function of DICER1 and miRNA. Credit: Addison Cooper, Cooper Graphics.

*DICER1* syndrome is a tumor predisposition syndrome caused by germline *DICER1* pathogenic variants. It typically presents in childhood. The *DICER1* gene encodes a micro-RNA-processing enzyme. Micro-RNA (miRNA) are short non-coding single-stranded RNA involved in numerous gene regulatory functions including silencing messenger RNA, impacting ribosome functioning, and chromatin remodeling, among other functions (Figure 1). Loss-of-function alterations of *DICER1* lead to defective miRNA and subsequent dysregulation of expression of many genes [1,2,3,4]. Individuals affected with *DICER1* syndrome are predisposed to benign and malignant neoplasms in multiple organ systems, and some surreptitiously feature macrocephaly. Outside the central nervous system, pleuropulmonary blastoma, multinodular goitre, cystic nephroma, and Sertoli–Leydig cell tumor of the ovary are the most common manifestations [5]. Within the central nervous system, metastatic pleuropulmonary blastoma, pituitary blastoma, embryonal tumor with multilayer rosettes (ETMR), and primary intracranial sarcoma, *DICER1*-mutant (PIS-*DICER1*), occur [6]. Although a relatively new diagnostic entity in the current WHO CNS classification scheme, PIS-*DICER1* appears to be a very rare and aggressive malignant brain tumor [7].

## 2. Case Presentation

**Figure 2:**
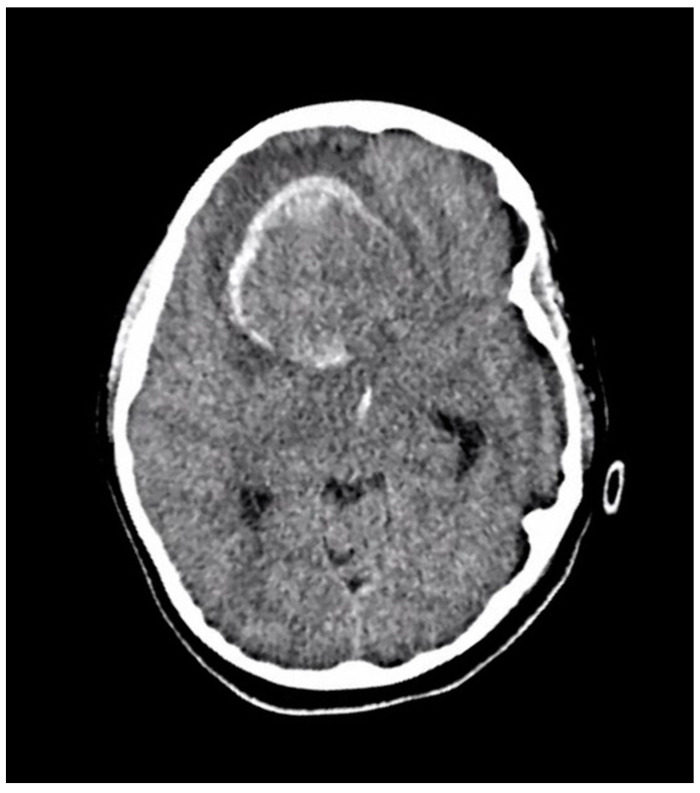
An axial section of a non-enhanced head CT scan performed in the emergency department at the time of acute presentation. It reveals a large well-defined right frontal mass with solid and hemorrhagic components. There is surrounding edema and midline shift.

A ten-year-old boy with a history of autism and macrocephaly presented acutely with altered level of consciousness, emesis, left hemiparesis, left facial droop, and slurred speech in the context of a four-week history of intermittent headache and vomiting. Computed tomography (CT) scan demonstrated a large right frontal intraparenchymal hemorrhage and suspected underlying intra-axial mass with intraventricular hemorrhagic extension, mass effect, and midline shift (Figure 2). The patient underwent emergency craniotomy for debulking of the lesion and tissue diagnosis.

The patient had no prior history of trauma, vascular disorders, or neoplasms. The patient’s family history included attention deficit hyperactivity disorder (ADHD), autism, and macrocephaly in several family members, including in two of the patient’s three siblings. There were intracranial neoplasms reported in several of the patient’s paternal second degree relatives, including meningioma, pituitary adenoma, and a non-specified intracranial malignancy. Additionally, he had third and fourth degree relatives with goitre and thyroid cancer. The patient’s ethnic background was Northern European and Slavic and his parents were non-consanguineous.

**Figure 3:**
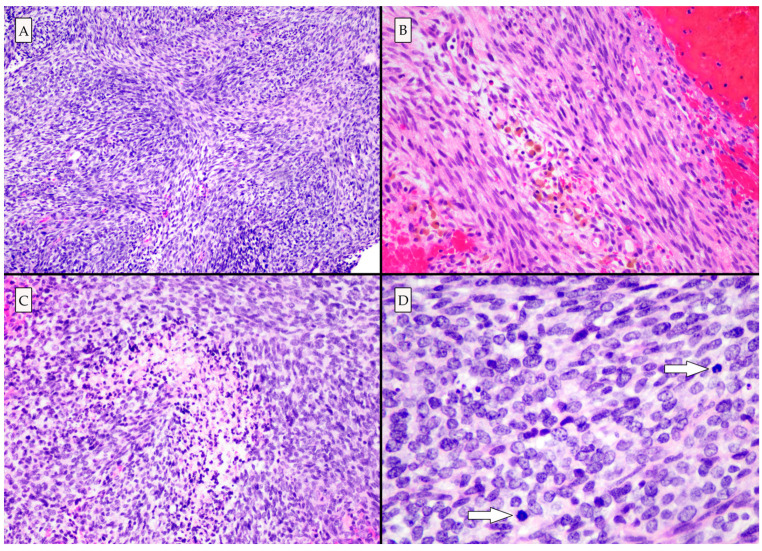
Photomicrographs of hematoxylin and eosin-stained slides. (**A**) The architecture is variable, though predominantly fascicular and storiform (100×). (**B**) Hemosiderin-laden macrophages are seen, indicating that the lesion had bled prior to the acute hemorrhagic presentation (200×). (**C**) High grade histologic features including necrosis (200×), and (**D**) mitotic activity (arrows) were present. There were areas of poorly differentiated small round blue cells (400×).

Histologic examination demonstrated a high-grade neoplasm with spindle cell and poorly differentiated small round blue cell morphologies (Figure 3). Eosinophilic globules and rhabdomyoblasts (which are commonly present in primary intracranial sarcoma, *DICER1*-mutant [8,9]) were not identified in this case. Immunohistochemical and histochemical studies revealed abundant intercellular reticulin deposition, strong vimentin and CD99 immunopositivity, and patchy desmin and myoD1 immunopositivity, in line with a mesenchymal neoplasm. Patchy nuclear H3K27Me3 and ATRX loss were identified [10].

Molecular testing of the lesion (Illumina Trusight pan-cancer RNA sequencing panel, Hospital of Sick Children, Toronto, Ontario) revealed the following: (1) a *DICER1* frame shift insertion (DICER1p.A1690Gfs*9), (2) a *DICER1* missense mutation (DICER1pE1813Q), and (3) a *KRAS* missense mutation (KRASp.G12L). Incorporating the light microscopic and genetic findings resulted in the final pathologic diagnosis of “primary intracranial sarcoma, *DICER1*-mutant”.

Germline testing (Invitae Diagnostic Testing) demonstrated a heterozygous *DICER1* frameshift variant (DICER1p.Ala1690Glyfs*10), identical to that appreciated on RNA sequencing. Parental germline testing for *DICER1* variants was negative, as was testing in one sibling (testing is pending for the other siblings). These results were suggestive of a de novo mutation in the patient, although the possibility of gonadal mosaicism was not entirely excluded, particularly considering the family history of macrocephaly, and intracranial and thyroid neoplasms.

Following his initial debulking surgery, the patient underwent disease staging, including magnetic resonance imaging (MRI) of the spine, whole-body positron emission tomography (PET) scan, bone marrow biopsy and aspirate, and cerebrospinal fluid cytology, all of which were negative for metastatic/distant sites of disease. One month following initial resection, he experienced a surgical site infection necessitating craniotomy and removal of the infected bone flap. Imaging at this time also revealed a recurrent mass lesion with rapid interval growth.

He was initiated on ICE (ifosfamide, carboplatin, and etoposide) chemotherapy consisting of ifosfamide 3 g/m^2^ IV on days 1 and 2, carboplatin 500 mg/m^2^ IV on day 3, escalating up to 600 mg/m^2^ based on tolerance, and etoposide 150 mg/m^2^ IV on days 1 and 2, given in 21-day cycles. Following six cycles, the patient had good radiographic response. He then went on to receive focal proton beam radiation to a dose of 60 cobalt gray equivalent (CGE) given in 30 fractions. He received a further two cycles of ICE, for a total of eight cycles, with no evidence of residual disease at the primary site at the end of therapy.

## 3. Discussion

Tumorigenesis in *DICER1* syndrome generally involves a multiple-hit mechanism. Most commonly, in addition to a germline loss of function mutation, tumors will possess a somatically acquired missense mutation often involving exon 24 or 25 encoding the RNase IIIb cleavage domain. These two “hits” do not fully prevent *DICER1* function and are thought to promote a tumorigenic environment due to the disruption of miRNA regulation of gene expression and acquisition of further oncogenic mutations. In addition to two *DICER1* pathogenic variants, additional oncogenic alterations are usually identified in *DICER1* related tumors, often involving *TP53* and/or the MAPK pathway [11]. In this case, A *KRAS* missense mutation was also identified. *KRAS* is an oncogene that encodes K-Ras, a GTPase that is a component of the MAPK pathway. *KRAS* mutations and amplifications are common molecular drivers in a variety of malignancies, including lung, colorectal, and pancreatic carcinomas, and leukemias [12,13].

Intracranial neoplasms are an uncommon manifestation of the *DICER1* syndrome, but nevertheless multiple types of intracranial tumors may be encountered. These include PIS-*DICER1*, ETMR, pineoblastoma, pituitary blastoma, and intracranial metastases of tumors from different sites [6]. The most recent WHO 2021 5th edition classification of central nervous system tumors recognizes PIS-*DICER1* as a new and distinct entity defined by the presence of *DICER1* mutations and its characteristic DNA methylation profile [7]. 

The nature of the relationship between *DICER1* syndrome and PIS-*DICER1* is an evolving question. In other neoplasms with *DICER1* mutations, the association with inherited *DICER1* syndrome is well-established. One study of 77 patients with pleuropulmonary blastoma and germline *DICER1* mutations showed that 13% of *DICER1* mutations appear de novo; the remaining 87% of cases showed germline *DICER1* mutations in parents. Mosaic phenotypes have also been described [11]. In PIS-*DICER1*, however, few documented cases arise in patients with a germline *DICER1* mutation and a typical *DICER1* syndrome phenotype. The literature on PIS-*DICER1* that includes patient and family germline *DICER1* analysis is uncommon. The first reported case of PIS-*DICER1* was in 2017 in a Peruvian child who did not possess a germline *DICER1* mutation [14]. In 2018, De Kock et al. reported a South American child with a 14q32.13q32.2 deletion (encompassing the *DICER1* gene) bearing a phenotype including cerebral sarcoma, ciliary body medulloepithelioma, lung cyst typical of pleuropulmonary blastoma type I/Ir, developmental delay, hypertonia, and facial dysmorphism [15]. Koelsche et al., 2018, performed germline testing on five patients with PIS-*DICER1*; germline *DICER1* loss-of-function mutations were identified in two of five patients [8]. Interestingly, neither of these two patients showed clinical evidence of a tumor predisposition syndrome at the time of diagnosis. Diaz Coronado et al., 2022, performed germline testing on 19 patients with PIS-*DICER1*; no germline *DICER1* mutations were identified in any of the 19 patients. Several of the patients in this study possessed cutaneous lesions and venous angiomas, and three of the patients fulfilled the clinical criteria for a diagnosis of neurofibromatosis 1 [16]. PIS-*DICER1* has been described in the context of neurofibromatosis 1 in one of three patients from a 2019 case series of PIS-*DICER1* [9]. The frequency of PIS-*DICER1* appears to be more in South American, particularly Peruvian, populations. The reason for the increased incidence in this demographic is unknown, although Diaz Coronado et al., 2022, proposed a hereditary predisposition and environmental factors [16].

As PIS-*DICER1* is a relatively newly described entity, first reported in 2017 [6], the literature around it has expanded rapidly. Since its first inclusion in the 2021 WHO classification of central nervous system tumors, more reports on PIS-*DICER1* have been published, refining understanding of the biology and treatment of these lesions. PIS-*DICER1* are most often supratentorial and aggressive tumors. Acute presentation (hours to days) with evidence of intratumoral bleed, similar to the case presented in this report, is a common initial manifestation [16,17]. There is no standard treatment for PIS-*DICER1*. A 2021 study of 68 patients with non-metastatic PIS-*DICER1* demonstrated that multimodal therapy including surgery, ICE chemotherapy, and radiotherapy lead to improved progression-free survival and overall survival compared to treatment with radiotherapy alone or chemotherapy and radiotherapy [16]. In this study, two-year progression-free survival ranged from 21 to 58% and two-year overall survival ranged from 46 to 71%. A 2015 Canadian study of 14 children with primary intracranial sarcomas similarly showed that gross total resection, ICE chemotherapy, and radiotherapy result in an encouraging survival rate [17]. A recent systematic review of *DICER1*-associated intracranial malignancies (including PIS-*DICER1*, pineoblastoma, ETMR, and pituitary blastomas) demonstrated that gross total resection and radiation therapy are associated with prolonged overall survival [18].

## 4. Conclusions

*DICER1* syndrome is a tumor predisposition syndrome caused by germline pathogenic alterations to the *DICER1* gene, which encodes a micro-RNA-processing enzyme. It is typically inherited in an autosomal dominant manner. Most cases are inherited, with de novo *DICER1* syndrome thought to be less frequent. The syndrome typically manifests in childhood with both benign and malignant neoplasms in multiple organ systems.

An uncommon manifestation of *DICER1* syndrome is PIS-*DICER1*. This high-grade intracranial sarcoma frequently shows unique histological features, in particular rhabdomyoblasts and eosinophilic globules, that might direct the pathologist to this underlying syndromic diagnosis [9]. However, such histologic features were absent in this case. This case highlights the importance of molecular diagnostics in intracranial sarcomatous tumors, especially in pediatric patients.

## Data Availability

Not applicable.

## References

[1] Foulkes W.D., Priest J.R., Duchaine T.F. (2014). DICER1: Mutations, microRNAs and mechanisms. Nat. Rev. Cancer.

[2] Hill D.A., Ivanovich J., Priest J.R., Gurnett C.A., Dehner L.P., Desruisseau D., Jarzembowski J.A., Wikenheiser-Brokamp K.A., Suarez B.K., Whelan A.J. (2009). DICER1 mutations in familial pleuropulmonary blastoma. Science.

[3] Kamihara J., Paulson V., Breen M.A., Laetsch T.W., Rakheja D., Shulman D.S., Schoettler M.L., Clinton C.M., Ward A., Reidy D. (2020). DICER1-associated central nervous system sarcoma in children: Comprehensive clinicopathologic and genetic analysis of a newly described rare tumor. Mod. Pathol..

[4] Pugh T.J., Yu W., Yang J., Field A.L., Ambrogio L., Carter S.L., Cibulskis K., Giannikopoulos P., Kiezun A., Kim J. (2014). Exome sequencing of pleuropulmonary blastoma reveals frequent biallelic loss of TP53 and two hits in DICER1 resulting in retention of 5p-derived miRNA hairpin loop sequences. Oncogene.

[5] González I.A., Stewart D.R., Schultz K.A.P., Field A.P., Hill D.A., Dehner L.P. (2022). DICER1 tumor predisposition syndrome: An evolving story initiated with the pleuropulmonary blastoma. Mod. Pathol..

[6] De Kock L., Priest J.R., Foulkes W.D., Alexandrescu S. (2020). An update on the central nervous system manifestations of DICER1 syndrome. Acta Neuropathol..

[7] WHO Classification of Tumours Editorial Board (2021). World Health Organization Classification of Tumours of the Central Nervous System.

[8] Koelsche C., Mynarek M., Schrimpf D., Bertero L., Serrano J., Sahm F., Reuss D.E., Hou Y., Baumhoer D., Vokuhl C. (2018). Primary intracranial spindle cell sarcoma with rhabdomyosarcoma-like features share a highly distinct methylation profile and DICER1 mutations. Acta Neuropathol..

[9] Lee J.C., Villanueva-Meyer J.E., Ferris S.P., Sloan E.A., Hofmann J.W., Hattab E.M., Williams B.J., Guo H., Torkildson J., Florez A. (2019). Primary intracranial sarcomas with DICER1 mutation often contain prominent eosinophilic cytoplasmic globules and can occur in the setting of neurofibromatosis type 1. Acta Neuropathol..

[10] Alexandrescu S., Meredith D.M., Lidov H.G., Alaggio R., Novello M., Ligon K.L., Vargas S.O. (2021). Loss of histone H3 trimethylation on lysine 27 and nuclear expression of transducin-like enhancer 1 in primary intracranial sarcoma, DICER1-mutant. Histopathology.

[11] Brenneman M., Field A., Yang J., Williams G., Doros L., Rossi C., Schultz K.A., Rosenberg A., Ivanovich J., Turner J. (2015). Temporal order of RNase IIIb and loss-of-function mutations during development determines phenotype in pleuropulmonary blastoma / DICER1 syndrome: A unique variant of the two-hit tumor suppression model. F100Research.

[12] Herdeis L., Gerlach D., McConnell D.B., Kessler D. (2021). Stopping the beating heart of cancer: KRAS reviewed. Curr. Opin. Struct. Biol..

[13] Timar J., Kashofer K. (2020). Molecular epidemiology and diagnostics of KRAS mutations in human cancer. Cancer Metastasis Rev..

[14] Alexandrescu S., Vargas S. DSS Case 2017-9 Cerebral Sarcoma. Proceedings of the 93rd Annual Meeting of Neuropathologists, Diagnostic Slide Session.

[15] De Kock L., Geoffrion D., Rivera B., Wagener R., Sabbaghian N., Bens S., Ellezam B., Bouron-Dal Soglio D., Ordóñez J., Sacharow S. (2018). Multiple DICER1-related tumors in a child with a large interstitial 14q32 deletion. Genes Chromosom. Cancer.

[16] Diaz Coronado R.Y., Mynarek M., Koelsche C., Mora Alferez P., Casavilca Zambrano S., Wachtel Aptowitzer A., Sahm F., von Deimling A., Schüller U., Spohn M. (2022). Primary central nervous system sarcoma with DICER1 mutation-treatment results of a novel molecular entity in pediatric Peruvian patients. Cancer.

[17] Lafay-Cousin L., Lindzon G., Taylor M.D., Hader W., Hawkins C., Nordal R., Laperriere N., Laughlin S., Bouffet E., Bartels U. (2016). Successful treatment of primary intracranial sarcoma with the ICE chemotherapy regimen and focal radiation in children. J. Neurosurg. Pediatr..

[18] Vuong H.G., Le M.K., Dunn I.F. (2022). A systematic review of the clinicopathological features and prognostic outcomes of DICER1-mutant malignant brain neoplasms. J. Neurosurg. Pediatr..

